# Fatty Acid Profile in Liver Tissue From Cattle Raised in Different Systems in the Eastern Amazon, Brazil

**DOI:** 10.1155/ijfo/5510620

**Published:** 2026-07-23

**Authors:** Andréa Viana da Cruz, Jamile Andréa Rodrigues da Silva, André Guimarães Maciel e Silva, Susana Paula Almeida Alves, Cristina Fernandes Xavier, Rui José Branquinho Bessa, Ana Paula Damasceno Ferreira, Adriny dos Santos Miranda Lobato, Elton Alex Corrêa da Silva, Thomaz Cyro Guimarães de Carvalho Rodrigues, Welligton Conceição da Silva, Raimundo Nonato Colares Camargo Júnior, Antônio Marcos Quadros Cunha, Vanessa Vieira Lourenço Costa, Leonel António Joaquim, Laíza de Kássia Mendes Conceição, Mariana Jucá Moraes, José de Brito Lourenço Júnior

**Affiliations:** ^1^ Institute of Veterinary Medicine, Federal University of Para—(UFPA), Brazilian Agricultural Research Corporation—Embrapa, Federal Rural University of Amazon—UFRA, Castanhal, Pará, Brazil; ^2^ Institute of Animal Health and Production, Federal Rural University of the Amazônia (UFRA), Belém, Pará, Brazil; ^3^ Interdisciplinary Research Center in Animal Health (CIISA), Faculty of Veterinary Medicine, University of Lisbon, Lisbon, Portugal, ulisboa.pt; ^4^ Faculty of Agronomy, Federal University of Pará, Cametá, Pará, Brazil, ufpa.br; ^5^ Health Science Institute, Federal University of Pará, Belém, Pará, Brazil, ufpa.br; ^6^ Angónia Zootechnical Station (EZA), Agricultural Research Institute of Mozambique (IIAM), Tete, Mozambique

## Abstract

Bovine liver is a food that is known to be nutritious and easily accessible to consumers. The nutritional quality of this food can be influenced by several factors, such as the rearing system. The objective of this study was to characterize the fatty acid profile in the liver tissue of cattle raised in the Brazilian Eastern Amazon, taking into account the effect of the rearing system and seasonality. Liver tissue samples from cattle raised in native pastures, in flooded areas, in cultivated pastures, in highlands and in a confinement, system was evaluated. The liver tissue from the extensive systems showed (*p* < 0.05) a higher proportion of saturated fatty acids (C16:0 and 17:0) and a higher proportion of monounsaturated fatty acids compared with the confinement liver tissue. The sum of polyunsaturated fatty acids was higher for the hepatic tissue of the feedlot system compared with the pasture system; however, the tissue of animals from the pasture system showed much higher percentages of long‐chain PUFA′s such as EPA, DHA, DPA, and their precursor, alpha‐linolenic acid. The information gathered is important for optimizing the production of beef and its offal with desirable nutritional properties for promoting human health.

## 1. Introduction

The level of intramuscular fat and the composition of fatty acids (FAs), along with the biological value of protein, trace elements, and vitamins, are important factors that contribute to the nutritional value [1, 2]. The liver is one of the largest bovine viscera, located in the abdominal cavity, with the function of metabolizing and storing nutrients [3]. Obtained during the slaughter process, it is known to be a source of food nutrients for the world population, rich in essential components of the diet, such as proteins, vitamin A, biotin, folic acid, and bioavailability of minerals [4]. Consuming 100 g of liver daily can fulfill up to 50% of the recommended intake for iron, zinc, selenium, B vitamins, and 100% of vitamin A [5]. In addition, it is well accepted by consumers due to its taste, ease of consumption, and affordable price.

Consumers are increasingly aware of the relationship between diet, health and wellness, resulting in healthier and more nutritious food choices. As a result, considerable attention has been paid to increasing beneficial FAs in animal products such as meat and milk [6].

Beef and other ruminant products are important dietary sources of conjugated linoleic acid (CLA), the most prominent of which is the cis‐9, trans‐11 isomer, which has been shown to contain several beneficial health‐promoting properties [7]. Essential FAs are also present in beef liver, which are not synthesized by the body and must be obtained from diet, and are important for human growth and development, with functions in homeostatic balance and structural components of cell membranes and tissues, with a requirement of polyunsaturated fatty acids (PUFAs) omega‐6 (n‐6) of 13 g/day and omega‐3 (n‐3) [8].

It is important to note that the FA profile of beef is influenced by several factors, such as genetics, sex, age, and also by the production system, in particular by the feed, where the use of concentrates, pastures, or different proportions of both have by far the greatest influence on the nutritional value of meat [9]. However, there are still gaps in the available information, especially in relation to beef liver in the raising systems of the Brazilian Eastern Amazon. Therefore, this study is aimed at characterizing the FA profile in the liver tissue of cattle from cattle rearing systems in the Eastern Amazon, Brazil.

## 2. Materials and Methods

### 2.1. Ethical Consideration

The study was conducted in accordance with the guidelines set forth by the Ethics Committee for the Use of Animals of the Federal Rural University of Amazonia (No. 1928240123), which permits the utilization of slaughtered animals.

### 2.2. Treatment and Animals

The animals were castrated and identified as mixed‐breed Nelore cattle from disparate rearing systems: Three were grazed, and one was confined. The age and weight of the animals exhibited variability according to the farming system, with ranges of 24–48 months for the first and 410–500 kg for the second (Table 1).

**Table 1:** General characteristics of each farming system.

Systems	Location	Rearing system	Food	Age (average in months)	Weight (average)
1	Santa Cruz do Arari (lat. 00°39 ^′^48 ^″^S, long. 49°10 ^′^30 ^″^W, alt.6 m	Traditional	Pastures, floodplains.	36–48	410 kg
2	Monte Alegre (lat.02°00 ^′^28 ^″^S, long. 54°04 ^′^09 ^″^W, alt.38 m)	Traditional	Native floodplain pastures.	36–48	410 kg
3	São Miguel do Guamá (lat. 01°37 ^′^36 ^″^ S, long.47°29 ^′^00 ^″^W, alt.10 m)	Cultivated pasture	Mombaça grass (*Megathyrsus maximus*), on dry land and supplemented with palm dregs (20 kg/animal/2 times a day)	24	500 kg
4	Confinement ‐ Santa Izabel do Pará (lat. 01°17 ^′^55 ^″^ S, long.48°09 ^′^38 ^″^ W, alt. 24 m)	Confinement	Soybean meal, barley, Mombaça silage (*Panicum maximum cv*. *Mombasa)*, corn meal, cassava peel, urea, high performance nucleus, and wheat germ inclusion in the rainy season (fed for 135 days).	24	500 kg

**Figure 1:**
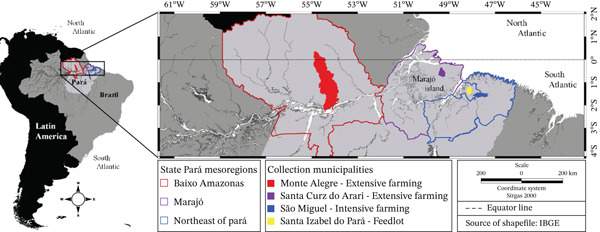
Location of the farm sampled.

The animals were sourced from a selection of properties within the Brazilian state of Pará. Two of the sampled properties were from traditional farming systems, one of which was located in the municipality of Santa Cruz do Arari, situated within the Marajó Archipelago, which falls within the Immediate Geographic Region of Soure‐Salvaterra [10]. The other was situated within the municipality of Monte Alegre, which falls within the Immediate Geographic Region of Santarém [10] (Figure 1). Their diet consisted of grazing on native pastures in floodplains and floodplain areas, respectively, at will.

The third property had a farming system based on cultivated pasture and was located in São Miguel do Guamá, a municipality in the immediate geographic region of Castanhal [10]. The feeding system consisted of ad libitum grazing of Mombasa grass (*Megathyrsus maximus*) and supplementation with palm dregs (20 kg/animal/2 times a day).

The final property selected was situated in the municipality of Santa Izabel do Pará, which is located in the immediate geographic region of Belém [10]. This property was the sole one to employ a confinement‐based breeding system. The feed provided to the animals in this system was based on soybean meal, barley, Mombaça silage (*Panicum maximum* cv. Mombaça), corn meal, cassava peel, urea, high‐performance nucleus, and the inclusion of wheat germ during the rainy season (Table 1).

### 2.3. Sample Collection and Preparation

Ninety‐six liver tissue samples were collected from cattle slaughtered in a commercial facility (12 per treatment per period; 24 per treatment overall). We followed the standards set by the Ministério da Agricultura, Pecuária e Abastecimento (MAPA), and we inspected everything in line with current legislation [11, 12]. We took the samples right after the animals were slaughtered, after the toilet stage. We collected 100 g of adipose tissue from each animal. We stored the samples in an ultrafreezer at −80°C until we could freeze‐dry them. We collected the animal tissues in two different seasons: one rainy season and one less rainy season. The first period was from January to June, and the second was from July to November.

We cut, ground, and froze the liver tissue samples in an ultrafreezer for 48 h at −80°C. They were then freeze‐dried nonstop for 48 h at −42°C to −45°C and a pressure of 158–256 *μ*m of Hg in a freeze‐dryer (LIOTOP, L101). They were then vacuum‐packed and sent to the Center for Interdisciplinary Research in Animal Health at the University of Lisbon for FA analysis.

### 2.4. FA Profile

The FA profile of the liver tissue was determined using the methyl ester derivative extraction method (FAMEs) by direct transesterification, according to [13]. The FAs were quantified by gas chromatography (Shimadzu 2010 Plus, Kyoto, Japan) and were identified by comparing the retention time of the methyl esters in the samples with FA standards.

The FAs were quantified by integrating the area under the methyl ester curve using the GS solution software (Shimadzu, Kyoto, Japan). The FA content was expressed as the percentage of total fatty acid (TFA) methyl ester quantified.

We calculated the FA concentrations by adding up the concentrations of the different types of FAs: saturated fatty acids (SFA), unsaturated fatty acids (UFA), monounsaturated fatty acids (MUFA), and PUFAs.

∑SFA,∑UFA,∑MUFA,∑PUFA.

We also looked at how the acid concentrations (∑*S* *F* *A*/∑*U* *F* *A*, ∑*M* *U* *F* *A*/∑*P* *U* *F* *A*) and omega‐6 and omega‐3 FAs are related.

The activity of the enzymes *Δ*9‐desaturase and elongase in adipose tissue was estimated using mathematical indices, according to Malau‐Aduli et al. [14]:

Δ916100−desaturase =C1619:cisC1619160:cis+C:.

Δ918100−desaturase = C1819:cisC1819180:cis+C:.

Elongase=100 C1801819:+C:cisC16016191801819:+C:cis+C:+C:cis.

The atherogenicity index (AI), as an indicator of cardiovascular disease risk, and the thrombogenicity index (TI), as an indicator of the onset of coronary thrombosis, were assessed according to Ulbricht and Southgate [15]:

AI=C1204160:+C140:+C:∑PUFA n−63+∑PUFA n−+∑MUFA.

TI=140:+160:+180:0.5×∑MUFA+0.56×∑PUFA n−+33×∑PUFA n−+∑PUFA n−36×∑PUFA n−.

### 2.5. Statistical Analysis

To analyze the data, the rearing systems and periods were compared using the non‐parametric Mann–Whitney *U* test.

Considering that each extensive system is in a different mesoregion (Santa Cruz do Arari–Marajó, Monte Alegre–Baixo Amazonas and São Miguel–Northeast Pará), at two times of the year (PC and PS), in addition to the confinement system (also in two periods), totaling eight treatments. The Kruskal–Wallis test was carried out to see if there were any differences between these treatments [16], and Dunn′s post hoc test to determine which of these treatments differed from each other [17]. Both procedures were carried out in the R software, via RStudio (Version 4.3.2) using the rstatix and stats packages, considering a significance level of 0.05.

A principal component analysis (PCA) was carried out in order to reduce the dimensionality of the data in a factorial plane, thus representing the original data in a multivariate space in two dimensions. To carry out the PCA, the matrix of FAs that represented more than 1% in at least one treatment was first transformed into log + 1, and then standardized. This standardization reduces the difference in FA percentages so that they all have the same relative importance in the calculation of the principal components. Both procedures were carried out in the R studio program Version 4.3.2. The standardization was carried out using the scale function, the PCA was carried out using the factorMiner package, and the biplot was visualized using the factoextra package.

## 3. Results

**Figure 2:**
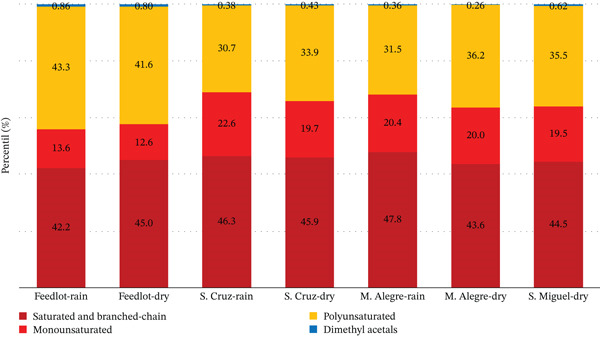
Percentage distribution of fatty acid classes (% of total FA) in the bovine liver tissue samples analyzed.

A total of 48 FAs were identified and quantified in bovine liver tissue. The mean values obtained for the FAs of the samples were grouped according to the degree of saturation. Figure 2 shows the macroanalysis values of the FAs present in bovine liver tissue. Proportionally dominated by SFA (47.8%), PUFAs (43.3%), with lower levels of MUFA (22.6%) and FAs derived from dimethylacetylation (DMA 0.86%).

For SFA, the highest concentration occurred in the Monte Alegre system in the wet season and the lowest concentration occurred in the feedlot in the wet season. For MUFA, the highest concentration was observed in Santa Cruz in the rainy season and the lowest in the confinement in the dry season. For PUFA, the highest concentration was observed in the Monte Alegre system in the dry season and the lowest in Santa Cruz in the rainy season.

The values presented in Table 2 were grouped according to the degree of saturation and only FAs contributing ≥ 1.0% in at least one treatment were reported.

**Table 2:** Fatty acid composition of cattle liver tissue (% by mass of total fatty acids).

	Extensive				São Miguel (intensive pasture)	Confinement (intensive)						SEM
	Santa Cruz		Monte Alegre					KW	MW			
	RS	DS	RS	DS	RS	RS	DS		$\begin{array}{c} Meal\times confinement \end{array}$	$\begin{array}{c} Extensive\times intensive\;\text{to}\;pasture \end{array}$	Period	
Saturated and branched‐chain FA												
C16:0	14.07 ab	14.95 a	11.50 ab	13.07 ab	10.37 bc	7.13 c	7.29 c	^***^	$\begin{array}{c} \text{Past}>\text{Con} \end{array}$ ^***^	$\begin{array}{c} \text{Ext}>\text{IP} \end{array}$ ^**^	$\begin{array}{c} \text{Dry}>\text{rain} \end{array}$ ^*^	1.05
C17:0	0.85 abc	0.94 a	1.04 a	0.87 ab	0.40 d	0.56 cd	0.68 bcd	^***^	$\begin{array}{c} \text{Past}>\text{Con} \end{array}$ ^***^	$\begin{array}{c} \text{Ext}>\text{IP} \end{array}$ ^***^	$\begin{array}{c} \text{Dry}>\text{rain} \end{array}$ ^*^	0.07
C18:0	29.25 bcd	28.15 cd	33.20 ab	28.08 d	32.62 abc	33.19 ab	36.07 a	^***^	$\begin{array}{c} \text{Con}>\text{past} \end{array}$ ^**^	$\begin{array}{c} \text{IP}>\text{Ext} \end{array}$ ^**^	ns	1.14

Monounsaturated FA												
C18:1t11	1.01 ab	1.30 a	0.99 ab	0.95 ab	0.87 bc	0.54 c	0.44 c	^***^	$\begin{array}{c} \text{Past}>\text{Con} \end{array}$ ^***^	$\begin{array}{c} \text{Ext}>\text{IP} \end{array}$ ^**^	ns	0.09
C18:1c9	18.26 a	14.88 ab	15.62 a	15.95 a	15.75 a	10.28 bc	9.37 c	^***^	$\begin{array}{c} \text{Past}>\text{Con} \end{array}$ ^***^	ns	$\begin{array}{c} \text{Rain}>\text{dry} \end{array}$ ^*^	0.98
C18:1c11	1.02 bcd	1.12 a	1.17 a	0.97 bcd	0.88 d	0.94 cd	1.04 bc	^*^	ns	$\begin{array}{c} \text{Ext}>\text{IP} \end{array}$ ^*^	ns	0.11

Polyunsaturated FA												
C18:2n‐6	8.96 bc	8.92 bc	6.60 c	7.74 c	14.57 ab	23.65 a	23.40 a	^***^	$\begin{array}{c} \text{Con}>\text{Past} \end{array}$ ^***^	$\begin{array}{c} \text{IP}>\text{Ext} \end{array}$ ^***^	ns	2.05
C18:3n‐3	2.49 a	1.66 ab	2.30 a	1.99 a	2.58 a	0.74 b	0.64 b	^***^	$\begin{array}{c} \text{Past}>\text{Con} \end{array}$ ^***^	$\begin{array}{c} \text{IP}>\text{Ext} \end{array}$ ^*^	$\begin{array}{c} \text{Rain}>\text{dry} \end{array}$ ^**^	0.28
C20:3n‐6	1.06 c	3.05 a	2.32 ab	2.89 ab	2.20 ab	2.30 ab	2.18 bc	^***^	ns	ns	$\begin{array}{c} \text{Dry}>\text{rain} \end{array}$ ^***^	0.24
C20:4n‐6	6.42 d	7.51 abc	6.92 cd	8.36 a	7.15 bcd	7.69 ab	7.49 abcd	^*^	ns	ns	$\begin{array}{c} \text{Dry}>\text{rain} \end{array}$ ^*^	0.38
C20:5n‐3	1.96 ab	2.13 ab	2.31 ab	2.43 a	1.37 bc	0.33 c	0.41 c	^***^	$\begin{array}{c} \text{Past}>\text{Con} \end{array}$ ^***^	$\begin{array}{c} \text{Ext}>\text{IP} \end{array}$ ^***^	ns	0.27
C22:4n‐6	0.45 d	0.77 cd	0.87 bc	0.81 bcd	0.90 bc	2.26 a	1.48 ab	^***^	$\begin{array}{c} \text{Con}>\text{Past} \end{array}$ ^***^	$\begin{array}{c} \text{IP}>\text{Ext} \end{array}$ ^*^	ns	0.19
C22:5n‐3	6.02 ab	6.74 a	6.56 a	8.13 a	4.55 bc	3.98 c	4.22 c	^***^	$\begin{array}{c} \text{Past}>\text{Con} \end{array}$ ^***^	$\begin{array}{c} \text{Ext}>\text{IP} \end{array}$ ^***^	$\begin{array}{c} \text{Dry}>\text{rain} \end{array}$ ^*^	0.52
C22:6n‐3	2.23 ab	2.18 ab	2.45 a	2.71 a	1.33 c	1.46 bc	1.36 c	^***^	$\begin{array}{c} \text{Past}>\text{Con} \end{array}$ ^*^	$\begin{array}{c} \text{Ext}>\text{IP} \end{array}$ ^***^	ns	0.21

*Note:* Pa > Co indicates the difference between the systems (Santa Cruz do Arari, Monte Alegre and São Miguel vs. confinement); IP > Ex indicates when intensive pasture is greater than extensive; Ex > IP indicates when extensive is greater than pasture intensive; season (RS × DS) indicates the difference between the periods (rainy × dry); drought > rain indicates when the dry season is longer than the rainy season; rain > drought indicates when the rainy season is longer than the dry period. The letters refer to Dunn′s post hoc test when the KW column is significant. The letters indicate differences in the means between the treatments. ≤ 0.001.

Abbreviations: Co, confinement; DS, dry season; Ex, extensive; IP, intensive on pasture; KW, Kruskal and Wallis test; ns, not significant; Pa, pasture; PM, Mann–Whitney test; RS, rainy season; SEM, standard error of the mean.

^*^≤ 0.05, ^**^ ≤ 0.01, ^***^≤ 0.001.

For SFA, palmitic FA (C16:0) showed a difference between systems and periods, with pasture systems being more abundant, especially extensive systems, with greater influence of the less rainy period. The highest percentages were observed in the extensive system of Santa Cruz, in both periods, the confinement system presented the lowest percentage in both periods analyzed.

For the heptadecanoic FA (C17:0), there was a difference between systems and periods, also in this case there was a greater influence of the pasture systems, where the extensive systems were greater than the intensive pasture systems. The highest percentages were observed in the native pasture systems of Santa Cruz and Monte Alegre, influenced by the less rainy season. The analyses for stearic FA (C18:0) showed that there was a difference between the systems, with the feedlot system being higher than the pasture systems; in this case, among the pasture systems, the São Miguel system presented a higher concentration. There was no difference in the periods analyzed. The lowest average was observed in the Monte Alegre system during the dry season.

The analyses for MUFA showed that for the vaccinated FA (C18:1,trans‐11), the pasture systems were more abundant than the feedlot, and in this case, the extensive systems presented a higher concentration, especially the Santa Cruz system, and the lowest average observed was in the feedlot system. No influence of the seasons was observed. The oleic FA (C18:1c9) in the pasture systems showed a difference between the confinement systems. The highest percentages were observed in Santa Cruz, Monte Alegre and during the rainy season in São Miguel. FA C18:1c11 did not show any difference between systems or for the period. There was a greater influence of the rainy season.

For PUFA, a higher abundance of linoleic (C18:2n‐6), arachidonic (C20:4n‐6), and docosapentaenoic (C22:5n‐3) FAs was observed. For linoleic FA, there was a difference between systems and periods, with the feedlot system being greater than the pasture systems, but in the analysis between pasture systems, the São Miguel system was more influential. And for the periods analyzed, there was no difference. For *α*‐linolenic FA (C18:3n‐3), a greater influence of the pasture systems was observed, mainly from São Miguel. The rainy season had the greatest influence.

The linolenic (C20:3n‐6) and arachidonic (C20:4n‐6) dihomo‐*γ*‐FA showed no differences between the systems. In both cases, the less rainy period was influenced. Regarding the eicosapentaenoicFA (C20:5n‐3), there was a difference between the systems, with the pasture systems being larger than the feedlot systems, with the emphasis on the traditional extensive systems. There was no difference in the time periods analyzed.

For adrenal FA (C22:4n‐6), there was a difference between the systems, with the feedlot system being larger than those raised on pasture, and for the periods analyzed, there was no difference.

For docosapentaenoic (DPA; C22:5n‐3) and docosahexaenoic (DHA; C22:6n‐3) FAs, there was a difference between the systems tested, with pasture systems being higher than feedlot, with higher percentages in traditional extensive systems. The analysis of periods showed no difference between the influence of the less rainy period for AG, DPA and DHA. Table 3 shows the results of the analysis of the sums and the nutritional indices.

**Table 3:** Ratios and indices of FAs in the hepatic tissue of cattle.

	Extensive				São Miguel (intensive per meal)	Confinement						SEM
	Santa Cruz		Monte Alegre					KW	MW			
	RS	DS	RS	DS	RS	RS	DS		$\begin{array}{c} Meal\times confinement \end{array}$	$\begin{array}{c} Extensive\times intensive\;\text{to}\;pasture \end{array}$	Period	
Partial sums (% total fatty acids)												
SFA	46.29	45.91	47.77	43.55	44.45	42.22	45.00	ns	$\begin{array}{c} \text{Pa}>\text{Co} \end{array}$ ^**^	$\begin{array}{c} \text{Ex}>\text{IP} \end{array}$ ^*^	ns	0.339
MUFA	22.04 a	19.00 a	19.91 a	19.44 a	19.02 a	13.34 b	12.35 b	^***^	$\begin{array}{c} \text{Pa}>\text{Co} \end{array}$ ^***^	ns	ns	0.423
*Cis*‐MUFA	19.74 a	16.43 ab	17.44 a	17.32 a	16.98 a	11.77 bc	11.07 c	^∗∗∗^	$\begin{array}{c} \text{Pa}>\text{Co} \end{array}$ ^***^	ns	ns	0.377
*Trans*‐MUFA	1.59 a	1.80 a	1.62 a	1.41 ab	1.37 ab	1.06 b	0.96 b	^***^	$\begin{array}{c} \text{Pa}>\text{Co} \end{array}$ ^***^	$\begin{array}{c} \text{Ex}>\text{IP} \end{array}$ ^*^	ns	0.044
PUFA	32.29 c	33.41 c	31.10 c	34.25 bc	34.68 abc	43.74 a	41.13 ab	^***^	$\begin{array}{c} \text{Co}>\text{Pa} \end{array}$ ^***^	$\begin{array}{c} \text{IP}>\text{Ex} \end{array}$ ^**^	ns	0.594
n‐6	17.16 c	20.69 bc	16.96 c	20.11 bc	25.14 ab	36.56 a	34.72 a	^***^	$\begin{array}{c} \text{Co}>\text{Pa} \end{array}$ ^***^	$\begin{array}{c} \text{IP}>\text{Ex} \end{array}$ ^***^	ns	0.860
n‐3	12.71 ab	12.72 ab	13.63 ab	15.27 a	9.84 bc	6.52 c	6.64 c	^***^	$\begin{array}{c} \text{Pa}>\text{Co} \end{array}$ ^***^	$\begin{array}{c} \text{Ex}>\text{IP} \end{array}$ ^***^	ns	0.396

Partial sums (mg/100 g fresh meat)												
SFA	15.09 abc	16.88 a	16.08 ab	19.27 a	13.50 bcd	13.47 cd	11.99 d	^***^	$\begin{array}{c} \text{Pa}>\text{Co} \end{array}$ ^***^	$\begin{array}{c} \text{Ex}>\text{IP} \end{array}$ ^*^	ns	0.379
TFA (mg/g DM)	90.31 bc	106.92 a	86.94 bc	98.02 ab	105.89 a	99.40 ab	82.64 c	^***^	$\begin{array}{c} \text{Pa}>\text{Co} \end{array}$ ^*^	$\begin{array}{c} \text{IP}>\text{Ex} \end{array}$ ^*^	ns	1.783
TFA (mg/g fresh meat)	43.35 a	26.93 abc	26.25 bc	22.01 c	33.42 ab	29.48 abc	24.16 c	^**^	ns	$\begin{array}{c} \text{IP}>\text{Ex} \end{array}$ ^*^	$\begin{array}{c} \text{Rain}>\text{dry} \end{array}$ ^**^	1.167
TL (%)	3.29 a	2.04 abc	1.99 bc	1.67 c	2.54 ab	2.24 abc	1.83 c	^**^	ns	$\begin{array}{c} \text{IP}>\text{Ex} \end{array}$ ^*^	$\begin{array}{c} \text{Rain}>\text{dr}{\text{y}}^{**} \end{array}$	0.089
n‐6	3.28 abc	3.09 abc	2.85 c	2.87 bc	3.47 ab	4.01 a	2.79 c	^*^	ns	$\begin{array}{c} \text{IP}>\text{Ex} \end{array}$ ^*^	ns	0.141
n‐3	5.25 a	3.37 a	3.58 a	3.40 a	3.28 a	1.93 b	1.60 b	^***^	$\begin{array}{c} \text{Pa}>\text{Co} \end{array}$ ^***^	ns	$\begin{array}{c} \text{Rain}>\text{dry} \end{array}$ ^*^	0.158
EPA‐DHA	1.73 a	1.23 ab	1.13 ab	1.15 ab	0.89 bc	0.53 c	0.43 c	^***^	$\begin{array}{c} \text{Pa}>\text{Co} \end{array}$ ^***^	$\begin{array}{c} \text{Ex}>\text{IP} \end{array}$ ^***^	ns	0.058
BCFA	0.61 a	0.28 a	0.32 a	0.20 ab	0.10 bc	0.11 bc	0.07 c	^***^	$\begin{array}{c} \text{Pa}>\text{Co} \end{array}$ ^***^	$\begin{array}{c} \text{Ex}>\text{IP} \end{array}$ ^***^	ns	0.031

Ratios and nutritional quality indices												
PUFA/SFA	0.714 bc	0.734 bc	0.651 c	0.77 abc	0.769 ab	1.082 a	0.911 a	^***^	$\begin{array}{c} \text{Co}>\text{Pas} \end{array}$ ^***^	$\begin{array}{c} \text{IP}>\text{Ex} \end{array}$ ^**^	ns	0.019
n‐6/n‐3	1.37 c	1.63 bc	1.26 c	1.32 c	2.57 ab	5.67 a	5.29 a	^***^	$\begin{array}{c} \text{Co}>\text{Pas} \end{array}$ ^***^	$\begin{array}{c} \text{IP}>\text{Ex} \end{array}$ ^***^	ns	0.201
h/H	3.63 bc	3.26 c	3.97 bc	3.92 bc	4.75 ab	7.06 a	6.82 a	^***^	$\begin{array}{c} \text{Co}>\text{Pas} \end{array}$ ^***^	$\begin{array}{c} \text{IP}>\text{Ex} \end{array}$ ^*^	ns	0.180
AI	0.27 ab	0.28 a	0.22 ab	0.23 ab	0.19 bc	0.13 c	0.13 c	^***^	$\begin{array}{c} \text{Pas}>\text{Co} \end{array}$ ^***^	$\begin{array}{c} \text{Ex}>\text{IP} \end{array}$ ^***^	ns	0.008
TI	0.76 bc	0.74 bc	0.75 bc	0.63 c	0.83 ab	0.93 ab	1.00 a	^***^	$\begin{array}{c} \text{Co}>\text{Pas} \end{array}$ ^***^	$\begin{array}{c} \text{IP}>\text{Ex} \end{array}$ ^*^	ns	0.018
*Δ*9‐desaturase C16	2.52 bc	2.48 bc	3.64 a	3.05 abc	3.25 ab	2.36 bc	2.06 c	^*^	$\begin{array}{c} \text{Pas}>\text{Co} \end{array}$ ^***^	ns	$\begin{array}{c} \text{Rain}>\text{Dry} \end{array}$ ^*^	0.096
*Δ*9‐desaturase C18	38.51 a	34.61 a	31.95 ab	36.25 a	32.57 ab	23.94 bc	20.63 c	^***^	$\begin{array}{c} \text{Pas}>\text{Co} \end{array}$ ^***^	$\begin{array}{c} \text{Ex}>\text{IP} \end{array}$ ^*^	ns	0.765
Elongase	77.00 bc	73.81 c	80.35 bc	76.44 c	81.85 ab	85.52 a	85.92 a	^***^	$\begin{array}{c} \text{Co}>\text{Pas} \end{array}$ ^***^	$\begin{array}{c} \text{IP}>\text{Ex} \end{array}$ ^**^	$\begin{array}{c} \text{Rain}>\text{dry} \end{array}$ ^*^	0.582

*Note:* Intensive and extensive indicate differences between systems (Santa Cruz do Arari, Monte Alegre, and $\text{S}\tilde{\text{a}}\text{o}\;\text{Miguel}>\text{feedlot}$); season, (RS > DS) indicates differences between periods (rainy and dry). The letters refer to Dunn′s post hoc test when the KW column is significant. Letters indicate differences in means between treatments.

Abbreviations: AI, atherogenicity index; cis‐MUFA, total cis monounsaturated fatty acids; DS, dry season; Ext, extensive; h:H, hypocholesterolemic/hypercholesterolemic ratio; Int, intensive; KW, Kruskal and Wallis test; MW, Mann–Whitney test; ns, not significant; PUFA, total polyunsaturated fatty acids; RS, rainy season; SEM, standard error of the mean; SFA, total saturated fatty acids; TI, thrombogenicity index; trans‐MUFA, total trans monounsaturated fatty acids.

^*^≤ 0.05, ^**^≤0.01, ^***^≤ 0.001.

For SFA, palmitic FA (C16:0) showed a difference between systems and periods, with pasture systems being more abundant, especially extensive systems, with greater influence of the less rainy period. The highest percentages were observed in the extensive system of Santa Cruz; in both periods, the confinement system presented the lowest percentage in both periods analyzed.

For the heptadecanoic FA (C17:0), there was a difference between systems and periods; also, in this case there was a greater influence of the pasture systems, where the extensive systems were greater than the intensive pasture systems. The highest percentages were observed in the native pasture systems of Santa Cruz and Monte Alegre, influenced by the less rainy season. The analyses for stearic FA (C18:0) showed that there was a difference between the systems, with the feedlot system being higher than the pasture systems; in this case, among the pasture systems, the São Miguel system presented a higher concentration. There was no difference in the periods analyzed. The lowest average was observed in the Monte Alegre system during the dry season.

The analyses for MUFA showed that for the vaccinated FA (C18:1,trans‐11), the pasture systems were more abundant than the feedlot, and in this case, the extensive systems presented a higher concentration, especially the Santa Cruz system, and the lowest average observed was in the feedlot system. No influence of the seasons was observed. The oleic FA (C18:1c9) in the pasture systems showed a difference between the confinement systems. The highest percentages were observed in Santa Cruz, Monte Alegre and during the rainy season in São Miguel. FA C18:1c11 did not show any difference between systems or for the period. There was a greater influence of the rainy season.

For PUFA, a higher abundance of linoleic (C18:2n‐6), arachidonic (C20:4n‐6), and docosapentaenoic (C22:5n‐3) FAs was observed. For linoleic FA, there was a difference between systems and periods, with the feedlot system being greater than the pasture systems, but in the analysis between pasture systems, the São Miguel system was more influential. And for the periods analyzed, there was no difference. For *α*‐linolenic FA (C18:3n‐3), a greater influence of the pasture systems was observed, mainly from São Miguel. The rainy season had the greatest influence.

The linolenic (C20:3n‐6) and arachidonic (C20:4n‐6) dihomo‐*γ*‐FA showed no differences between the systems. In both cases the less rainy period was influenced. Regarding the eicosapentaenoicFA (C20:5n‐3), there was a difference between the systems, with the pasture systems being larger than the feedlot systems, with the emphasis on the traditional extensive systems. There was no difference in the time periods analyzed.

For adrenal FA (C22:4n‐6), there was a difference between the systems, with the feedlot system being larger than those raised on pasture, and for the periods analyzed, there was no difference.

For docosapentaenoic (DPA; C22:5n‐3) and docosahexaenoic (DHA; C22:6n‐3) FAs, there was a difference between the systems tested, with pasture systems being higher than feedlot, with higher percentages in traditional extensive systems. The analysis of periods showed no difference between the influence of the less rainy period for AG, DPA and DHA. Table 3 shows the results of the analysis of the sums and the nutritional indices.

The total proportion of *Σ*PUFA n‐6 was affected by the rearing system, with the confinement system showing a higher concentration because the intensive system was greater than the extensive; this is different from what was observed in the proportion of *Σ*PUFA n‐3, where the extensive system was greater than the intensive system, represented mainly by Monte Alegre. There was no difference in the periods for *Σ*PUFA n‐6 and *Σ*PUFA n‐3. The proportions of cis‐MUFA and trans‐MUFA were influenced by the extensive system. In this case, the periods were not significant.

In the fresh part of the samples, the SFA content was influenced by the grazing systems, especially by the extensive systems, with no influence of the periods. For the analysis of TFA in dry matter, the pasture systems presented a higher level than the feedlot, different from that observed in the fresh portion, which did not present a difference between these systems, although in both cases the São Miguel system presented a greater contribution.

The total lipids (LT) and n‐6 present in the fresh samples also showed no difference between the pasture and feedlot systems, but in the analysis between the pastures, São Miguel presented a greater contribution. In the analysis of the n‐3 content in the fresh portion, the pasture systems showed the greatest contribution and showed no differences between them. The pasture systems were the ones that contributed the most to the levels of EPA‐DHA and branched‐chain fatty acids (BCFAs) in the fresh portion, with emphasis on the extensive systems. There was no effect of season.

The estimated means for the n‐6/n‐3 and PUFA/SFA ratios were significant for the influence of the housing systems in both cases. The liver tissue of the confinement system showed higher levels of PUFA/SFA, n‐6/n‐3, and h/H. In the analysis between the pasture systems, the São Miguel system showed a greater influence. Regarding the periods, no significant influence was observed.

The evaluation of the AI and TI showed that there was a difference between the systems. In this case, the pasture system influenced the AI, especially São Miguel, and the confinement system influenced the TI. There was no difference for both periods analyzed.

The desaturation indices C16 and C18 were significantly influenced by the grazing systems, especially in the Monte Alegre and Santa Cruz systems, respectively. The rainy season influenced the activity of the C16 desaturase enzyme, with the rainy season being longer than the dry season, and for the C18 desaturation index there was no influence of the periods. The activity index of the enzyme elongase was influenced by the rearing system, with higher values in the rearing system.

**Figure 3:**
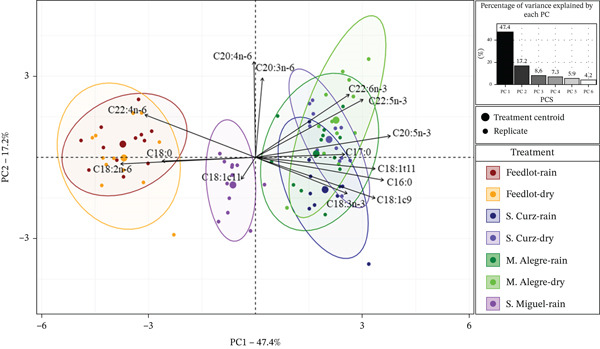
Analysis of the main components of fatty acids in the liver tissue of cattle.

PCA (Figure 3) explained 63.3% of the variation in FA in bovine liver tissue. PC1 explained 45.1% and PC2 explained 18.2%. Of the 14 GAs that were above 1% in at least one treatment, seven were representative of the variation between treatments.

In the negative part of PC1, the confinement system was isolated in both periods, where a higher concentration of PUFAs of the omega‐6 group (C22:4n‐6 and C18:2n‐6) was observed. In the positive part of PC1, the Monte Alegre and Santa Cruz systems were isolated in both periods, where a higher concentration of SFA (C16:0), MUFA (C18:1c9), and PUFA of the omega‐3 group (C20:5n‐3 and C22:5n‐3) could be observed.

In the positive part of PC2, the vector of PUFA C20:4n‐6 is indicated, where Monte Alegre obtained higher concentrations in the dry period. Inversely proportional to PC2 in the negative part are São Miguel in the dry season and Santa Cruz in the rainy season.

## 4. Discussion

This study presents an unprecedented approach, considering that there are no studies that demonstrate the results obtained in the context of the Brazilian Eastern Amazon for liver tissue of beef cattle.

The confinement system, by presenting lower concentrations of palmitic (16:0) and heptadecanoic (17:0) FA, and those of Santa Cruz and Monte Alegre with the lowest concentrations of stearic FA (18:00), present a positive aspect for the consumer from a nutritional point of view, since these acids are considered hypercholesterolemic because they interfere with the normal function of low‐density lipoprotein receptors (LDLR), reducing its removal and increasing its concentration in plasma [18]. The lower concentration of C16:0 in the liver of confined animals is probably due to the conversion of C16:0 into C18:0 mediated by the enzyme elongase, which showed a greater activity index in this group of animals. Guerra et al. [19] and Turkyilmaz et al. [20] found the same relationship between elongase enzyme activity and C16:0 concentration. Fiorentini et al. [21] also reported a greater elongase enzyme activity index and a lower concentration of C16:0 in the muscle and adipose tissue of feedlot Nellore cattle, receiving leenssed oil as a source of dietary lipids.

On the other hand, although the intensive rearing system showed a higher concentration of C18:0, unlike the others mentioned above, C18:0 FA is known to have a neutral effect on blood cholesterol concentrations because this FA is rapidly converted to oleic acid (18:1cis9), which has hypocholesterolemic effects [22].

Palmitic SFA (16:0) has properties that increase low‐density lipoprotein (LDL) and HDL cholesterol [23]. High intake of these acids increases blood cholesterol levels [23], and diets high in saturated fat are thought to contribute to the development of heart disease, weight gain, and obesity. The expression of LDLR in hepatocytes is primarily responsible for blood cholesterol levels and depends on the activity of the enzyme hydroxymethylglutaryl coenzyme A (HMG‐CoA) reductase, an enzyme essential for the intracellular synthesis of hepatic cholesterol [24].

The profile of FAs in cattle liver tissue indicated that the extensive systems had higher levels of MUFA, particularly oleic acid (18:1c9). This is different from what was previously reported by Daley et al., [25] who reported that grain fed beef produced higher levels of this group of FAs.

The PUFA group contains the most important FAs in terms of disease prevention, as there is evidence that linoleic acid (18:2 n‐6) and alpha‐linolenic acid (18:3 n‐6) are essential for humans and that the risk of coronary heart disease is reduced when PUFA replace SFA [23].

The highest bioactivity is observed for long‐chain n‐3 PUFAs, such as eicosapentaenoic acid (EPA; 20:5 n‐3), docosapentaenoic acid (DPA; 22:5 n‐3), and docosahexaenoic acid (DHA; 22:5 n‐3), which have been shown to have beneficial effects in the prevention of heart attack, depression, and cancer [25, 26]. In this study, the liver tissue of cattle showed a higher concentration of PUFA than that observed by Nogales et al. [27], who characterized the lipid profiles in the Longissimus thoracis muscle of male and female Marismeña cattle raised in the wild or in captivity under intensive conditions.

The beneficial effects of the longer chain n‐3 PUFA, eicosapentaenoic acid (EPA; 20:5 n‐3) and docosahexaenoic acid (DHA; 22:6 n‐3), in reducing the risk of cardiovascular disease, cancer, and Type 2 diabetes, and their critical role in proper brain function, fetal visual development, and lifelong maintenance of neural and visual tissues are well established [28, 29]. In this study, the extensive rearing system had a greater influence on the results for the PUFA n‐3 group compared with the intensive system.

Forages contain high proportions of *α*‐linolenic acid (18:3n‐3), so animals fed forage‐based diets may have meats richer in omega‐3 FA compared with animals fed concentrates [30]. However, the meat of these animals also contains trans‐linked intermediates resulting from the metabolism of 18:3n‐3 in the rumen, such as 18:1trans‐11, 18:2trans‐11, cis‐15, and especially CLA‐cis‐9, trans11, which is mostly biosynthesized from 18:1trans‐11 in tissues by the action of the enzyme stearoyl Co‐A desaturase (SCD). Thus, the enrichment of CLA‐cis‐9, trans‐11 in meat implies a high substrate availability, a high SCD activity and a high deposition of IMF, as discussed by Bessa et al. [30].

In general, meat produced by animals under extensive conditions is leaner, with IMF rich in n‐3 PUFA, CLA, and t11‐18:1, which are related to health maintenance [25].

It is important to mention CLA, which is a collective term that includes several conjugated isomers of linoleic acid, such as rumenic acid (18:2‐c‐9, t‐11) and 18:1t‐10, c‐11, formed during the biohydrogenation process in the rumen, which is a group associated with the prevention of inflammation, cancer, atherosclerosis, obesity, diabetes, and osteoporosis, among other benefits for human health [31]. The CLA content in animal products (meat and milk) is strongly influenced by the consumption of fresh pastures [32]. This fact is supported by the results, as differences were observed between extensive and intensive systems.

It is important to note that foods of animal origin, such as ruminants, can have high levels of C18:1t10 or 18:1t11, and when enriched sources are considered, C18:1t10 has properties similar to industrial trans AG, which is highly detrimental to human health and a possible promoter of cardiovascular disease, whereas T 11‐18:1 can be converted to a conjugated linoleic acid isomer, both of which have potential positive health effects. In addition, increases in C18:1 t10 in meat‐producing ruminants have not been associated with adverse effects on meat quality. However, at high levels, it may affect the nutritional quality of beef [33].

The PUFA/SFA ratio is an important way to evaluate the nutritional value of red meat, and according to Scollan et al. [34], the recommended value should be above 0.5. Comparing the mentioned value with the results of this study, it can be seen that the analyzed liver tissues are in accordance with the recommended and indicated values for human consumption, since all were above 0.5, with emphasis on the PUFA/SFA ratio of the confinement system.

The n‐6/n‐3 PUFA ratio is an important metric because it reflects the nutritional value and optimal ratio of n‐6/n‐3 omegas in the human diet and is associated with a significant reduction in the risk of coronary heart disease [35].

The n‐6/n‐3 ratio for beef is beneficially low (typically < 3), reflecting the significant amounts of desirable n‐3 PUFA, especially *α*‐linolenic acid (18:3 n‐3), but also EPA, docosapentaenoic acid (DPA; 22:5 n‐3), and DHA. The analysis of beef has shown values of the omega‐6/omega‐3 ratio between 1.5 and 10.4. Some authors have reported higher values of the n‐6/n‐3 ratio compared with this study. Higher levels of n‐6/n‐3 are generally found in feedlot animals and the lowest levels are found in meat from cattle raised on pasture [36], in this context the observed results are consistent with the literature, as higher levels were observed in the intensive system and lower levels in the extensive system.

Newly formed FAs in the liver can be desaturated and elongated [37]. In the case of desaturases, they influence several important biological properties of FAs, including membrane fluidity [38], antioxidant activity [39], and inflammatory processes [40]. In beef, the most abundant MUFA is oleic acid (C18:1), a result of metabolic processes of desaturase and/or elogas enzymes from stearic acid (C18:0) [41]. According to St John et al. [37], the capacity for desaturation of FAs in the liver of cattle is limited, in addition, adipose tissue is the main site where elongation and desaturation of FAs by the enzyme *Δ*‐9‐desaturase occurs. In addition, the activity index of the enzyme *Δ*‐9‐desaturase 18 and elongase takes into account the oleic acid content in the tissue, and in this study both the concentrations of this FA and the activity were higher in the extensive system.

The h/H index represents the ideal ratio between hypercholesterolemic and hypocholesterolemic FAs, and for meat products a value of 2 is considered [42]. These indices are important because they allow a better nutritional assessment, indicating whether or not the food has a harmful potential for human health, inferring the risk of coronary and cardiovascular diseases, such as atherosclerosis and stroke [43]. Considering that in this study the h/H ratios are higher than the mentioned value, the analyzed liver tissues can be considered unsuitable for consumption and cardiovascular health.

The study of the lipid quality indices of atherogenicity (AI) and thrombogenicity (TI) takes into account the different effects that FAs can have on human health and, in particular, the probability of increasing the incidence of atherosclerosis and/or thrombosis; although there are no recommended values for these indices, meats with low TI and IA values can be considered of better nutritional quality for humans [44]. In this context, the farming systems influenced the reduction of AI and TI rates, with the intensive system having a lower AI and the extensive systems having a lower TI.

## 5. Conclusion

In conclusion, the analyses of the FA profile in cattle liver tissue indicated the influence of the housing system on the FA profile and, in addition, significant differences between the systems and little difference for the seasonal periods. The overall profile for SFA indicated that the liver tissue of the pasture systems is not as healthy compared with the feedlot, although the highest ratio of SFA to the feedlot liver tissue is composed of 18:0, rather than other FAs that are considered hypercholesterolemic. PUFAs are the FAs that present the highest protective bioactivity, and in this research, compared with the liver tissue obtained in the feedlot system, the tissue of animals from pasture systems showed much higher percentages of these FAs, particularly the most representative long‐chain PUFAs, such as EPA, DHA, and DPA, and their precursor, alpha‐linolenic acid. The information gathered is important for optimizing the production of beef and its offal with desirable nutritional properties for the promotion of human health.

## Funding

This study was supported by the Centro Interdisciplinar de Investigação em Sanidade Animal (UIDB/00276/2020), Associated Laboratory for Animal and Veterinary Science (LA/P/0059/2020), and LEAF (UIDB/04129/2020).

## Conflicts of Interest

The authors declare no conflicts of interest.

## Data Availability

The data supporting the findings of this study can be accessed openly on 10.5281/zenodo.17902538.
